# Comparing high altitude treatment with current best care in Dutch children with moderate to severe atopic dermatitis (and asthma): study protocol for a pragmatic randomized controlled trial (DAVOS trial)

**DOI:** 10.1186/1745-6215-15-94

**Published:** 2014-03-26

**Authors:** Karin B Fieten, Wieneke T Zijlstra, Harmieke van Os-Medendorp, Yolanda Meijer, Monica Uniken Venema, Lous Rijssenbeek-Nouwens, Monique P l’Hoir, Carla A Bruijnzeel-Koomen, Suzanne GMA Pasmans

**Affiliations:** 1 Department of (Pediatric) Dermatology and Allergology, Wilhelmina Children’s Hospital, University Medical Center Utrecht, Heidelberglaan 100, 3584 CX, Utrecht, The Netherlands; 2 High altitude clinic Merem Dutch Asthma Center Davos, Herman-Burchardstrasse 1, CH-7265 Davos, Switzerland; 3 Department of Pediatrics, Onze Lieve Vrouwe Gasthuis, Oosterpark 9, 1091 AC Amsterdam, The Netherlands; 4 Department of Pediatric Pulmonology and Allergology, Wilhelmina Children’s Hospital, University Medical Center Utrecht, Lundlaan 6, 3584 EA, Utrecht, The Netherlands; 5 Department of Pediatric Psychology, Wilhelmina Children's Hospital, University Medical Center Utrecht, Lundlaan 6, 3584 EA, Utrecht, The Netherlands; 6 TNO Prevention and Health, Zernikedreef 9, 2333 CK Leiden, The Netherlands; 7 Department of Pediatric Dermatology, Sophia Children’s Hospital and KinderHaven, Erasmus University Medical Center, Wytemaweg 80, 3015 CN Rotterdam, The Netherlands

**Keywords:** Atopic dermatitis, Atopic eczema, Asthma, Atopic syndrome, Children, Coping, Quality of life, High altitude treatment, Multidisciplinary treatment, RCT

## Abstract

**Background:**

About 10 to 20% of children in West European countries have atopic dermatitis (AD), often as part of the atopic syndrome. The full atopic syndrome also consists of allergic asthma, allergic rhinitis and food allergy. Treatment approaches for atopic dermatitis and asthma include intermittent anti-inflammatory therapy with corticosteroids, health education and self-management training. However, symptoms persist in a subgroup of patients. Several observational studies have shown significant improvement in clinical symptoms in children and adults with atopic dermatitis or asthma after treatment at high altitude, but evidence on the efficacy when compared to treatment at sea level is still lacking.

**Methods/Design:**

This study is a pragmatic randomized controlled trial for children with moderate to severe AD within the atopic syndrome. Patients are eligible for enrolment in the study if they are: diagnosed with moderate to severe AD within the atopic syndrome, aged between 8 and 18 years, fluent in the Dutch language, have internet access at home, able to use the digital patient system Digital Eczema Center Utrecht (DECU), willing and able to stay in Davos for a six week treatment period. All data are collected at the Wilhelmina Children’s Hospital and DECU. Patients are randomized over two groups. The first group receives multidisciplinary inpatient treatment during six weeks at the Dutch Asthma Center in Davos, Switzerland. The second group receives multidisciplinary treatment during six weeks at the outpatient clinic of the Wilhelmina Children’s Hospital, Utrecht, the Netherlands. The trial is not conducted as a blind trial. The trial is designed with three components: psychosocial, clinical and translational. Primary outcomes are coping with itch, quality of life and disease activity. Secondary outcomes include asthma control, medication use, parental quality of life, social and emotional wellbeing of the child and translational parameters.

**Discussion:**

The results of this trial will provide evidence for the efficacy of high altitude treatment compared to treatment at sea level for children with moderate to severe AD.

**Trial Registration:**

Current Controlled Trials ISRCTN88136485.

## Background

About 10 to 20% of children in West European countries are diagnosed with atopic dermatitis (AD) [1]. AD and allergic asthma (AA) are the most common chronic diseases in children. They can be considered different manifestations of the atopic syndrome which also includes allergic rhinitis and food allergy [2]. It has been estimated that 30 to 36% of the children diagnosed with AD aged years or younger will be diagnosed with asthma at age 6 years or older [3]. The reverse has also been demonstrated; in a cohort of children diagnosed with asthma aged between six and nine years, 20% had developed AD after nine years of follow-up [4]. The majority of children with AD are currently treated on an outpatient basis. Treatment approaches include topical treatment with emollients, anti-inflammatory therapy with topical immunosuppressive-like agents (corticosteroids and calcineurin inhibitors), anti-microbial treatment and educational programs, according to current guidelines [5,6].

However, symptoms persist in a subgroup of children with moderate to severe AD. Often these children have an extensive treatment history and are unable to gain sufficient control of their disease. It has been shown that a multidisciplinary approach to AD treatment is beneficial for children who do not respond well to regular treatment, and often results in sustained clinical improvement [7,8]. The multidisciplinary model includes medical and psychosocial evaluation and treatment, and educates the children and their parents using a stepwise approach to the management of AD [8]. Psychosocial evaluation is important because children have to learn to cope with the specific problems associated with living with a chronic disease [9]. Furthermore, they have to learn to adhere to strict medicinal regimes, which are time consuming. Often they are restricted in activities, such as sports, swimming or outside play. Social and emotional problems may occur because of the distinguished appearance and characteristics of AD [10]. Sleep deprivation due to nightly symptoms of itch may lead to tiredness, mood changes and impaired psychosocial functioning of the child and his/her family [10]. AD has a significant negative impact on the quality of life of children and their parents. With increasing AD severity, reported quality of life decreases further [11,12].

Since the 1950s Dutch children with asthma are referred to the Dutch Asthma Center in Davos, located at 1560 m altitude in the Swiss Alps, for ‘high altitude’ treatment [13]. Important characteristics of the alpine climate are the dry air, the low exposure to allergens (such as house dust mite and fungi), and the increased exposure to UV radiation [13]. There is also a relative lack of pollution [14]. Several observational studies have shown significant improvement in quality of life and clinical symptoms (Fev1 (forced expiratory volume in 1 second), FeNO (fraction of exhaled nitric oxide) and SCORAD (scoring atopic dermatitis) among others) in children and adults with AD and asthma after treatment at high altitude [15-21]. However, no randomized trials have been done to investigate the superiority of high altitude treatment compared to treatment at sea level in improving quality of life or disease activity in children with AD or asthma.

In the DAVOS trial, we compare multidisciplinary treatment at high altitude and sea level in a pragmatic randomized design. The objective of this study is to find the most effective treatment strategy for children with difficult to treat AD. This paper describes the study design and the interventions.

## Methods/Design

### Design and setting

A pragmatic randomized controlled trial was designed in collaboration with the Department of Pediatric Dermatology/Allergology in the Wihelmina Children’s Hospital (a national referral center in the Netherlands for children with moderate to severe AD and the atopic syndrome) and the high altitude clinic Dutch Asthma Center Davos in Switzerland. Dutch children with moderate to severe AD are either treated during six weeks at the Dutch Asthma Center Davos (inpatient treatment) or at the Wilhelmina Children’s Hospital (outpatient treatment). All children are expected to be included in the study within a time period of four years.

**Figure 1:**
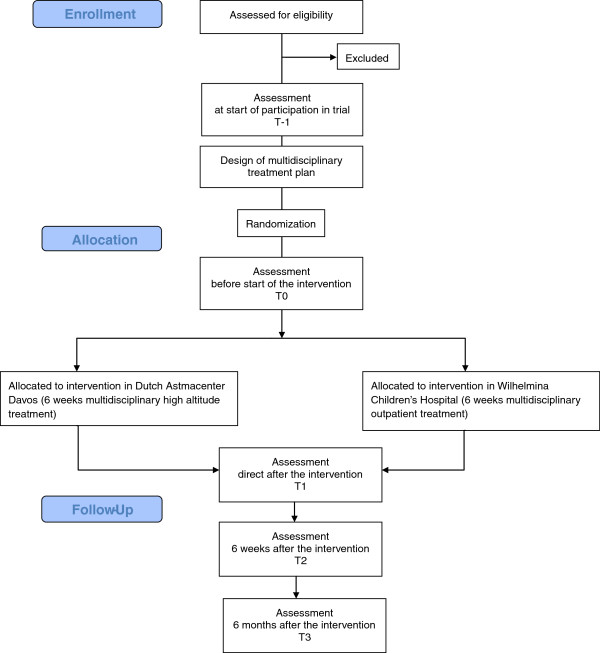
Flowchart of study design.

The trial consists of three different components: psychosocial, clinical and translational. The children in the study will be extensively monitored on these three aspects at study inclusion and during the study period. The study period includes the actual enrollment in the study, a six week intervention period and follow-up measurements at six weeks and six months after the intervention (Figure 1). Enrollment and all follow-up measurements will be done at the Wilhelmina Children’s Hospital. The Digital Eczema Center Utrecht (DECU) will be used for data collection and communication [22]. Within this digital portal possibilities for e-consulting, self-management training and online monitoring are combined.

### Ethical considerations

This study follows the Dutch Medical Research Involving Human Subjects Act 1998 (WMO) and the Helsinki Declaration principles 2008, meaning that the legal representatives of all participants will sign a written informed consent, stating that participation can be withdrawn at any time without any negative consequences concerning their current or future medical treatment. All study procedures have been reviewed and approved by the Medical Ethics Committee of the Utrecht Medical Center, the Netherlands (reference 09-192/K).

### Participants

The DAVOS trial is designed for children in the Netherlands with difficult to treat AD who are unable to gain sufficient control of their disease with current treatment strategies. Children are eligible for enrolment in the study if they are: diagnosed with moderate to severe AD within the atopic syndrome; aged between 8 and 18 years; fluent in the Dutch language; have internet access at home; able to use the Digital Eczema Center Utrecht; and willing and able to stay in Davos for a six week treatment period.

Children are not eligible for enrolment if they are currently participating in another study.

### Recruitment, inclusion procedure and consent

The DAVOS trial is a national trial conducted in the Netherlands. Children with moderate to severe AD will be recruited from the outpatient clinic of the Wilhelmina Children’s Hospital and from other hospitals in the country. Dutch dermatologists, pediatricians, allergists, general practitioners and patient support groups were informed about the trial in journals, conferences and using online media.

Children and/or their parents who are interested in the trial can register themselves directly in the DECU. A questionnaire with items regarding allergic complaints, use of (topical) immunosuppressive therapy such as corticosteroids, and more specific questions regarding AD activity (including a self-administered eczema area and severity index (SA-EASI)), questions on nightly complaints of itch and sleeping behavior and, if relevant, questions on asthma, rhinitis and food allergy complaints has to be answered in order to register. Possible study participants are invited by the dermatologist to the outpatient clinic and assessed for eligibility. They are not required to stop or change their medication at this point. Children who fulfill the inclusion criteria are then informed about the study. If they are interested in participating in the trial, they are invited for a complete evaluation by the multidisciplinary team at the Wilhelmina Children’s Hospital, and informed consent is signed.

### Sample size

The sample size calculation for the psychosocial part is based on the primary outcome coping with itch, measured with the JUCKKI, children’s itching cognitions questionnaire [23]. A difference of five points is expected between the groups on the subscale catastrophisation (negative thoughts that have gone out of control). This difference was based on a study by Staab *et al.* on the effect of educational programs in children and adolescents with AD [24]. In this study, the SD of both groups was 7.6.

An additional sample size calculation was carried out for the clinical part of the trial. This was based on the primary outcome disease activity, measured with the SA-EASI [25]. Based on the study by Staab *et al.*, in which SCORAD was used to measure disease activity, a difference of 10 points between the groups and a SD of 15 for each group was expected [24,26].

With a power of 80%, 36 children are needed in each group. Assuming a drop-out rate of 10%, 40 children will be assigned to each of the two study conditions.

### Randomization

Children are randomly assigned to either intervention or control groups. Randomization is done in SAS (statistical analysis system) with a custom made program by an external data manager using a covariate-adaptive randomization method, controlled for age and diagnosis of asthma [27].

### Multidisciplinary treatment

The multidisciplinary team consists of a dermatologist, a pediatric allergist or pediatrician, psychologist and (dermatology/research) nurse. Weekly multidisciplinary evaluation during the intervention between the involved professional teams in Utrecht and Davos takes place using video conference. In this way, expertise is being combined and optimal care for all included patients is guaranteed. A short summary of discussed topics is provided in the patient record in the DECU.

Before randomization and start of the intervention a multidisciplinary treatment plan is developed, with input from both multidisciplinary teams, to make sure the treatment goals can be achieved under either intervention and control conditions. Based on the intake (history of received treatment, psychological questionnaires and intelligence outcomes), specific problem areas are identified and each healthcare professional writes down the treatment goals and strategy in a treatment plan. Psychosocial as well as clinical treatment goals are formulated. Examples of treatment goals are: adherence to treatment; influence and acceptance of AD in everyday life, including distinguished appearance; correct application of topical corticosteroids; coping with itch; sleep disturbances; and knowing what to do during an exacerbation. Planned interventions to achieve the goals are specified, as well as the responsible healthcare professional. There are no treatment restrictions during the trial. The treatment plan is discussed with the child and their parents and may be modified if needed. External professionals who are already involved with the child, for example the referring physician, nurses or social workers, are invited to participate during the study through the DECU, with permission of the child and their parents. In this way, optimal continuation of transmural treatment for the patient is made possible after the end of the study in the home region of the patient.

### The intervention in Davos

Children are admitted to the Dutch Asthma Center Davos for a period of six weeks in groups of four children. The children attend a school integrated into the clinic. There are also social workers who accommodate the group, and each child has his/her own mentor.

The treatment program consists of several fixed elements. Every day, a group physical activity is organized under supervision of a physiotherapist, such as fitness, swimming or outdoor activities. Weekly individual treatment sessions take place with the pediatrician, the psychologist alternating with the psychomotor therapist, and the physiotherapist (if needed). The nurse monitors correct application of topical treatment in individual sessions with each child twice daily. In these individual sessions health education is provided with a varying weekly theme. The content of the individual consultations depends on the problems described in the treatment plan. Children and their parents are separated during the six week treatment period, however parents usually visit once.

### Control condition in Utrecht

Recently, a new multidisciplinary intervention has been developed for children with AD combining education, involvement of both dermatologists, pediatricians, psychologists and nurses, and the use of the DECU. Children are seen during half a day at the outpatient clinic on a weekly basis for a period of six weeks. During this period they have three consultations with the dermatologist, five consultations with the dermatological nurse, three consultations with the pediatric allergist and three consultations with the psychologist. The content of the individual consultations depends on the treatment goals described in the treatment plan.

In the third week a group medical appointment (also known as group consultation) is scheduled for the children and their parents separately, about coping with AD and compliance [28]. It is a substitute for an individual appointment with a clinician during which children and their parents discuss their personal experiences and advise and support each other.

A description of intervention and control conditions is provided in Table 1.

**Table 1:** Overview of control and intervention conditions

	**Treatment location**	
	**Wilhelmina Children's Hospital**	**Dutch Asthma Center Davos**
Multidisciplinary team	Dermatologist, pediatric allergist, psychologist, dermatology nurse	Pediatrician, psychologist, nurse, psychomotor therapist, physiotherapist, social workers
Treatment program	Three individual consultations with dermatologist, pediatrician and psychologist	Six individual consultations with pediatrician, four with the psychologist, three with the psychomotor therapist
	One group consultation (children and parents separate)	Physical activity program under supervision of physiotherapist
Educational program	Five individual consultations with the dermatology nurse	Twice daily individual consultations with the dermatology nurse
Treatment setting	Outpatient	Inpatient
Environment	Own home in the Netherlands	Clinic in Swiss alpine environment

### Follow-up period

There is a six month follow-up period during which all children are assessed three times at the Wilhelmina Children’s Hospital: immediately after the intervention, six weeks after the intervention and six months after the intervention, which is the end of the follow-up period. During the entire follow-up period, children are encouraged to use the self-management tools in the DECU. In case of questions and/or exacerbations, they can contact the dermatology nurse with an e-consultation and, if needed, an appointment with the dermatologist is made. All children are encouraged to play sports, for example at FitKids, a Dutch organization that facilitates physical activity programs for children with a chronic disease under supervision of a children’s physiotherapist [29].

### Primary and secondary outcomes

The primary outcomes of this study are coping with itch and quality of life of the child for the psychosocial part and disease activity for the clinical part. Coping levels are measured with the JUCKKI questionnaire for children (aged 8 to 12 years) and the JUCKJU questionnaire for adolescents (aged 13 to 18 years) [23]. Quality of life is assessed with the CDQLI questionnaire [30]. Disease activity is assessed with the SA-EASI [25].

Secondary outcomes for the psychosocial part include social and emotional wellbeing of the child, feelings of autonomy and fear, quality of life and stress levels of the parents.

Secondary outcomes for the clinical part include medication use, TARC (thymus and activation-regulated chemokine), total and specific IgE (Immunoglobulin E), asthma control parameters such as FeNO (fraction of exhaled nitric oxide), ACQ (asthma control questionnaire), PAQLQ (pediatric asthma quality of life questionnaire), BHR (bronchial hyperresponsiveness) and spirometric variables such as FEV1 (forced expiratory volume in 1 second), FVC (forced vital capacity) and their ratio (FEV1/FVC). During the follow-up period, the number of flares, the number of dermatologist/pediatric/psychologist appointments, e-consultations and emergency visits or hospital admissions are recorded.

At each time point in the study, questionnaires are filled in by the child and his/her parents, blood samples are taken from the child, and lung function is assessed. A detailed overview of all parameters and measurements is provided in Table 2.

**Table 2:** Study assessments

**Assessment**	**Measured with**	**T-1**	**T0**	**T1**	**T2**	**T3**
Coping with itch and disease	JUCKKI-COPECI (8-12 yr) or JUCKJU-COPEJU (13-18 yr) [23]	x	x	x	x	x
Disease-specific quality of life	CDQLI [31]	x	x	x	x	x
Disease activity and control (AD)	EASI [33]	x	x	x	x	x
	SA-EASI [25]	x	x	x	x	x
	Used topical corticosteroids	x	x	x	x	x
	TARC [34]	x	x	x	x	x
Disease activity and control (Asthma)	ACQ [35]	x	x	x	x	x
	PAQLQ [36]	x				x
	Lung function test	x	x	x	x	x
	FeNO	x	x	x	x	x
	Used medication	x	x	x	x	x
	Metacholine provocation	x	x	x	x	x
Serum	Total IgE		x			x
	Specific IgE: inhalation/food: ImmunoCAP		x			x
	Specific IgE: inhalation/food: ImmunoCAP ISAC		x			
	Eosinophils		x	x	x	x
	Cytokine profile		x	x	x	x
Questionnaires	Social demographic information	x				
	Intelligence test [37]	x				
	SF-36 [38,39]	x				
	CBCL [40,41]	x				
	Corticosteroid use	x			x	x
	NPV-J [42]	x				
	TRF [40]	x				
	CBSK/CBSA [43,44]	x		x		x
	ZBV-K [45]	x	x	x		x
	PUL (>12 yr) [46]	x		x		x
	Quality of life (parents) [47]	x	x	x		x
	ZBV [48]	x		x		x
	NOSI-K [49]	x		x		x
	NOSI-events [49]	x				x
Other	Maximal cycle ergometer test	x				x
	Bacterial colonization of the skin		x	x	x	x
	Bacterial colonization of the nose		x	x	x	x
	Skin strips for protease activity		x	x	x	x

ACQ, asthma control questionnaire; CBCL, child behavior checklist; CBSK, self-perception profile for children; CBSA, self-perception profile for adolescents; CDQLI, the children’s dermatology life quality index; COPEKI/COPEJU, coping with disease questionnaire; EASI, eczema area and severity index; FeNO, fraction of exhaled nitric oxide; IgE, Immunoglobulin E; ImmunoCAP, ImmunoCAP system (Thermo Fisher Scientific, Uppsala, Sweden); ImmunoCAP ISAC, Immunosolid-phase allergen chip (Thermo Fisher Scientific, Uppsala, Sweden); JUCKKI/JUCKJU, itching cognitions questionnaire; NOSI-events, parental stress index-events; NOSI-K, parental stress index- short form; NPV-J, Dutch personality questionnaire-youth version; PAQLQ, pediatric asthma quality of life questionnaire;

PUL, positive outcome list; SA-EASI, self-administered eczema area and severity index; SF-36, short form health survey; TARC, thymus and activation-regulated chemokine; T-1, assessment at start of participation in trial; T0, assessment before start of the intervention; T1, assessment direct after the intervention; T2, assessment six weeks after the intervention; T3, assessment six months after the intervention; TRF, teacher report form; ZBV, state-trait anxiety inventory; ZBV-K, state-trait anxiety inventory for children.

### Data collection and data analysis

In this study two sources of electronic data are used. The DECU gives a detailed overview of the intervention and follow-up period and contains the data from the questionnaires, assessments and e-consultations. Additional patient data is extracted from the electronic patient file in the Wilhelmina Children’s Hospital. After data collection, all data will be combined into one SPSS (Statistical Product and Service Solutions) database.

Data will be analyzed on an intention to treat basis. To examine whether there is a significant difference on the primary outcomes between the intervention and control group, a mixed-effects regression model will be used. Fixed effects for time, intervention group and the time*group interaction will be included in the model in order to examine the differences between the groups over time. To adjust for possible curvilinearity a squared term for time will be included in the model. A random intercept and random slope per child will be included in order to account for repeated measures within children. Baseline values of the outcome variables are included as fixed covariates to examine their possible impact on the observed treatment effect.

Data relating to asthma outcomes will be analyzed separately for all children diagnosed with asthma. All analyses conform to a specified plan. There are no interim analyses or stopping rules.

## Discussion

The DAVOS trial is a pragmatic randomized controlled trial that investigates the effect of multidisciplinary treatment at high altitude compared to sea level on coping, quality of life and disease activity in children with moderate to severe AD within the atopic syndrome. It is designed as a pragmatic trial, which fits best with our objective to find a new intervention with optimal treatment results for children with difficult to treat AD [31]. In this study we compare inpatient treatment in a high altitude clinic in Switzerland with outpatient treatment in a national expert center in the Netherlands. We chose for outpatient treatment in the Wilhelmina Children’s Hospital, because it currently offers the most specialized and intensive treatment in the Netherlands for this group of patients. It is most efficient to organize the involved medical disciplines to see the patients consecutively in an outpatient setting. Furthermore, we want to evaluate the best treatment for children with difficult to treat AD that minimizes the burden but maximizes the result. This means that if it is possible to reach significant clinical improvement with an intense six week outpatient program in the Netherlands, this is the preferred choice compared to a six week inpatient treatment in the Netherlands. Also, it would not be possible to extrapolate results from inpatient treatment in an academic hospital compared to a peripheral hospital. We believe that in this trial design, we are comparing the best options for treatment that are also likely to be carried out in practice after the end of the trial.

There are several differences between the intervention and control conditions, such as the differences in setting: inpatient and outpatient, own environment and change to alpine environment. During the intervention in Davos, adherence to treatment is more likely to increase due to the clinical setting. Children are separated from their parents which can be perceived as an extra burden for the child. It may also be an advantage, especially when the coping strategy of the child is negatively influenced by the coping strategies of the family [32].

All children in both interventions are discussed in weekly videoconferencing sessions with both multidisciplinary teams. AD treatment in both interventions is supervised by a single dermatologist in the Netherlands to minimize differences. Corticosteroid use is reduced or increased according to similar stepwise plans in both treatment arms. The educational plan is co-developed by both centers and consists of similar elements in both treatment arms. It is administered by the nurse and there is frequent contact between the nurses of both treatment arms.

The pragmatic nature of our trial will not allow us to find out how or why the interventions work, or which elements of the interventions contribute most to the observed treatment effects. Therefore we will study effectiveness: the long-term benefit of either treatment in routine clinical practice [31].

This study has several strengths. Children that participate come from all over the Netherlands. They often have an extensive treatment history and still experience an impaired quality of life, despite treatment according to current guidelines. In this study, children receive multidisciplinary treatment with individualized treatment goals either in their own environment or in an alpine clinical environment. The results of this trial will show which would be the most appropriate approach to provide treatment that results in sufficient long-term disease control for children with difficult to treat AD. The findings of our trial will be directly applicable in practice in the Netherlands.

The study also has potential drawbacks. It is an intense period for the study participants in which they have to visit the Wilhelmina Children’s Hospital several times for the scheduled assessments. Each assessment means a missed day of work for the parents and a missed school day for the child. This may lead to a larger drop-out rate than anticipated and it may take a lot of effort to keep the study participants motivated for all assessments. In this study neither the study participants nor the healthcare professionals assessing outcomes were blinded to the received intervention. This may cause bias for the more subjective outcome measures, such as the SA-EASI.

To the best of our knowledge, this is the first randomized trial that compares high altitude treatment with treatment at sea level in children with difficult to treat AD. Our study provides new and detailed information on the characteristics of these children. We hope to provide evidence for an intervention that gives the best results in terms of coping, quality of life and disease activity for this group of children.

## Trial status

The first patient was included in the study in September 2010. At the moment patient recruitment and data collection is in progress. We expect to complete patient recruitment by May 2014.

## Abbreviations

AA: Allergic asthma; ACQ: Asthma control questionnaire; AD: Atopic dermatitis; BHR: Bronchial hyperresponsiveness; CDQLI: Children’s dermatology quality of life index; DECU: Digital Eczema Center Utrecht; FeNO: Fraction of exhaled nitric oxide; FEV1: Forced expiratory volume in one second; FVC: Forced vital capacity; JUCKKI/JUCKJU: Itching cognitions questionnaire; PAQLQ: Pediatric asthma quality of life questionnaire; (SA)-EASI: (Self-administered) Eczema area and severity index; SAS: Statistical analysis software; SCORAD: Scoring atopic dermatitis; SD: Standard deviation; TARC: Thymus and activation-regulated chemokine.

## Competing interests

The authors declare that they have no competing interests.

## Authors’ contributions

KF: conception and design, data collection and analysis, manuscript writing and final approval of the manuscript. WZ: conception and design, data collection and analysis, manuscript writing and final approval of the manuscript. HO: conception and design, critical revision and final approval of the manuscript. YM: data collection, critical revision, final approval of the manuscript. MUV: data collection, critical revision, final approval of the manuscript. LR: data collection, critical revision, final approval of the manuscript. MH: conception and design, critical revision, final approval of the manuscript. CBK: conception and design, critical revision and final approval of the manuscript. SP: conception and design, data collection, critical revision and final approval of the manuscript. All authors read and approved the final manuscript.
